# Mouse Embryonic Retina Delivers Information Controlling Cortical Neurogenesis

**DOI:** 10.1371/journal.pone.0015211

**Published:** 2010-12-08

**Authors:** Ciro Bonetti, Enrico Maria Surace

**Affiliations:** Telethon Institute of Genetics and Medicine (TIGEM), Naples, Italy; Dalhousie University, Canada

## Abstract

The relative contribution of extrinsic and intrinsic mechanisms to cortical development is an intensely debated issue and an outstanding question in neurobiology. Currently, the emerging view is that interplay between intrinsic genetic mechanisms and extrinsic information shape different stages of cortical development [1]. Yet, whereas the intrinsic program of early neocortical developmental events has been at least in part decoded [2], the exact nature and impact of extrinsic signaling are still elusive and controversial. We found that in the mouse developing visual system, acute pharmacological inhibition of spontaneous retinal activity (retinal waves-RWs) during embryonic stages increase the rate of corticogenesis (cell cycle withdrawal). Furthermore, early perturbation of retinal spontaneous activity leads to changes of cortical layer structure at a later time point. These data suggest that mouse embryonic retina delivers long-distance information capable of modulating cell genesis in the developing visual cortex and that spontaneous activity is the candidate long-distance acting extrinsic cue mediating this process. In addition, these data may support spontaneous activity to be a general signal coordinating neurogenesis in other developing sensory pathways or areas of the central nervous system.

## Introduction

Spontaneous activity (SA) influences critical developmental events in different neuronal developing circuits such as the retina, the cochlea, the spinal cord and other brain structures [3], [4]. In particular, developing sensory organs, prior to acquiring the capability to transform physical stimuli in patterns of neuronal activity, generate bursts of action potentials that convey key information for sensory circuitry assembly [5]. Connectivity among relaying neurons from the periphery to the cortex provides the scaffold necessary to transfer the coordinated cues that shape the sensory systems as a whole [6]. In early postnatal life the visual system retinal SA (i.e. retinal waves; RWs [7] is essential for segregation and refinement of retinotopic maps in subcortical and cortical targets and is necessary for the development of various receptive field properties [5], [8]. However, the role of RWs during embryonic stages of visual system development at the time when connectivity emerges between the retina, the thalamus, and the cortex is unknown. Cholinergic RWs initiate in the mouse retina around embryonic day 16 (E16) [9], and disappear around the time of eye-opening [5]. Cholinergic retinal waves (sometimes called Stage 2) start at P1 and end at P10. Later in development (P10–P13, Stage 3), waves are mediated by a glutamatergic circuit and are not affected by nAChR antagonists. Earlier in development (Stage 1), waves are partially mediated by cholinergic transmission, thus blocking nAChRs inhibits but not the retinal waves [5], [9], [10]. Remarkably, RWs are transmitted to the cortex and drive early patterns of spontaneous activity (spindle burst) in neonatal V1 cortex in rats [11], [12].

The onset of RWs in mice and other species [4], [5] correlates spatially and temporally with three processes of visual system development, i) targeting of the main retinal output to the dorsal Lateral Geniculate Nuclei (dLGN) [13], ii) invasion of the sub-plate (SP) of the developing cortex by ingrowing dLGN thalamic axons [14], and iii) peak of layer IV neurogenesis [15]. Are these correlated spatio-temporal events also causally linked? Here, we set out to explore whether spontaneous activity arising from the embryonic retina and propagating along the nascent visual pathways connection contains long-distance information capable of modulating cell genesis in the developing visual cortex.

## Materials and Methods

### Animal procedures

Animal studies were performed in accordance with experimental protocols approved by Institutional Animal Care and Use (IACUC); ethics committee/institutional review board: Dipartimento Sanità Pubblica Veterinaria Dir. Gen. della Sanità Animale e del Farmaco Veterinario, Ufficio VI Benessere Animale Ministero della Salute (Ministry of Health). Approval ID: A3442, entitled: “***Terapia genica di malattie retiniche in modelli animali***” Application date 05-17-2007.

Fetal intravitreal injections of 0.3 ml of epibatidine (1 mM, Sigma, St. Louis, Mo., USA), forskolin (10 mM, Sigma, St. Louis, Mo., USA), NKH477 (10 mM, water soluble analogous of Forskolin, Tocris, UK), PBS (vehicle for epibatidine) or DMSO 100% (vehicle for forskolin) were performed with pregnant C57BL6 N on embryonic day late E15.5. Embryonic day 0.5 (E0.5) corresponded to midday of the day of the vaginal plug. Ex-utero surgery was performed as described previously [16] . To determine the position of the micropipette tip and accuracy of the injections, the drugs or vehicles were combined with 0.025% (w/v) fast green (Sigma, St. Louis, Mo., USA). Monocular enucleation (ME) was performed by first inserting a micropipette in the developing eye in order to favor the holding and then the removal of the eyeball by tweezers. Four hours after the closure of the abdominal wall, BrdU 50 mg/kg was administered. Twenty-four hours or 4 days after drug delivery, cervical dislocation was used to sacrify the mothers for prenatal and P0 studies. Cholera Toxin B – Alexa Fluor 594 (0.3 µl; Molecular Probes) of a 2 mg/ml solution in PBS was injected, concomitantly to epibatidine (1 mM) into the fetal eyes with the same procedures described.

### Immunohistochemistry

Immunohistochemical analyses were performed as described previously [1]. Briefly, frozen sections 12 µm were boiled in 10 mM sodium citrate, pH 6.0, and blocked in 10% normal goat serum (NGS, sigma) and 0.3% Triton for 1 hr at room temperature. Incubation with primary antibodies was performed at 4°C overnight. Secondary antibodies were applied to the sections for 2 hr at room temperature. The primary antibodies utilized were as follows: rat anti-Ki67 (1∶50, DAKO), mouse anti-BrdU (1∶300, Sigma) and anti-NF (165 KDa; 1∶100 DSHB). Secondary antibodies were conjugates of Alexa Fluor 488, 594 (1∶1000, Invitrogen). Finally, slices were washed and mounted in Vectashield with DAPI (4′,6′ – diamidino-2-phenylindole) (Vectstain), which was used as nuclear counterstaining.

### RNA *in situ* hybridization

*In situ* hybridization was performed on sectioned brains, which were cryoprotected by treatment with 30% sucrose in PBS and embedded in optimal cutting temperature compound (OCT; Miles, Elkhart, IN). Twenty-micrometer adjacent sections were hybridized overnight at 65°C with the Rorβ digoxigenin-labeled sense and antisense riboprobes [1]. The Rorβ probes (kindly provided by Dr. M. Studer) were obtained by linearizing the plasmid containing the Rorβ coding sequence transcribed with either T7 RNA polymerase (antisense probe) or T3 RNA polymerase (sense probe) [17].

### Quantification and statistical analysis

In the immunostaining experiments, the fluorescence cells were acquired with a light-sensitive charge-coupled device (CCD) digital camera DFC350 FX (Leica, Germany). At least 3 images for specimen were taken on serial adjacent sections. To quantify fluorescence cells, the counts were done in blind. All the data were analyzed and graphs were constructed using Microsoft Excel software. Error bars represent the standard error of the mean (s.e.m.). Statistical significance was determined using two-tailed Student's t-tests, two samples equal variants.

## Results and Discussion

**Figure 1:**
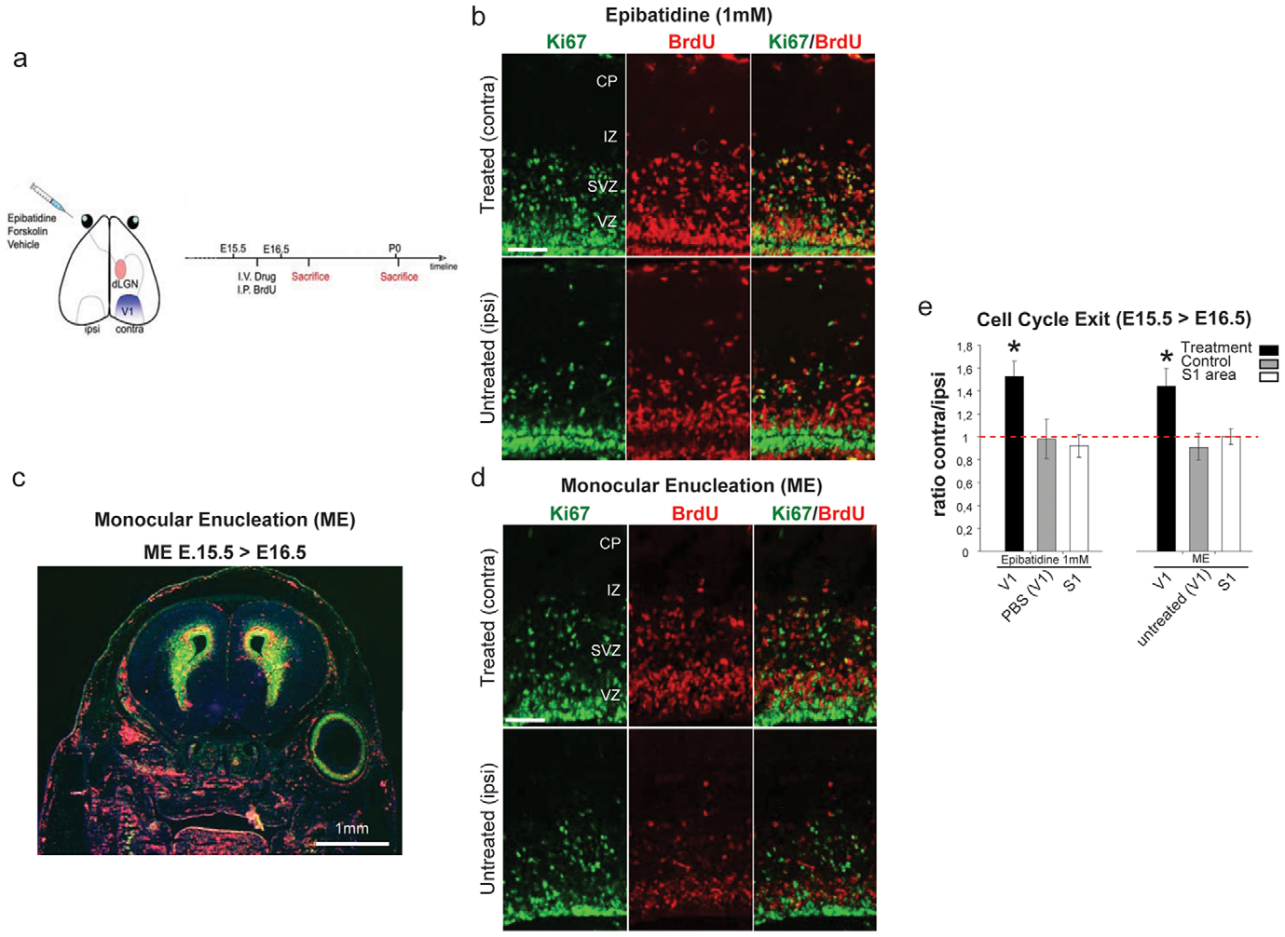
Inhibition of fetal retinal spontaneous activity and Monocular Enucleation (ME) increases corticogenesis. (**a**) Schematic representation of the intraocular pharmacological injection to evaluate the effect of the treatment on the contralateral (treated) compared to ipsi-lateral (untreated) developing visual (V1) or somatosensory cortex (S1). The intravitreal (I.V.) injection of epibatidine or monocular enucleation (ME) were performed at E15.5 (supplementary video S1, Figure 1) and BrdU was intraperitoneally (I.P.) administered 4 h later. (**b** and **d**) Representative images of Ki67 (green, left panels), BrdU (red, middle panels) and merged labelling (right panels) in contra-lateral (top) and ipsi-lateral (bottom) cortices after epibatidine treatment (**b**) or ME (**d**) of E16.5 embryos. Cells withdrawn from the cell cycle are BrdU+/Ki67−. Cells re-entering the cell cycle are BrdU+/Ki67+. (**c**) Representative image of Ki67 (green) BrdU (red) staining of an E16.5 embryonic head monocularly enucleated at E15.5. (**e**) Quantification of cell cycle exit rate, reported as the ratio between contra- and ipsi-lateral cortices, in E16.5 embryos upon administration of epibatidine or ME. Epibatidine treatment or ME (epibatidine n = 6, PBS n = 4 p = 0.0004; ME n = 4, untreated n = 3 p = 0.01, Student's t-test, two samples equal variants) increased neurogenesis. None of the treatments resulted in changes of neurogenesis in somatosensory areas (S1, epibatidine on V1 vs S1 n = 4 p = 0.0033; ME on V1 vs S1 n = 4 p = 0.01, Student's t-test, two samples equal variants). Abbreviations: CP cortical plate, IZ intermediate zone, SVZ Sub-ventricular zone, VZ ventricular zone.

**Figure 2:**
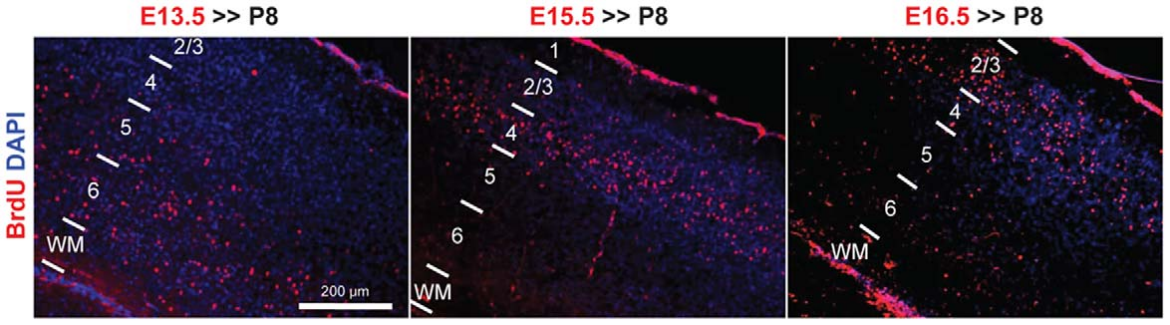
Layer-specific corticogenesis in the V1 cortex. Representative images of BrdU staining on P8 visual cortices of untreated mice that received BrdU injection at E13.5 (left), E15.5 (middle) and E16.5 (right), respectively. The sections were counterstained with DAPI. The cells labeled (BrdU+) at the time of ocular treatments (E15.5) were mainly located in visual layer IV of terminally layered cortex (P8).

To perturb stage I cholinergic RWs, we used a pharmacological paradigm [18]. We injected murine fetuses monocularly at E15.5 [16] with epibatidine (nicotinic acetylcholine receptor, nAChR agonist; 1 mM) to inhibit RWs firing pattern. To study the rate of neurogenesis of visual cortical progenitors [19] we counted the fraction of cells that had exited the cell cycle 24 hours after the S-phase marker 5-bromo-2′-deoxyuridine administration (BrdU, injected 4 hours after fetal surgery; Fig. 1, supplementary Fig. S1 and supplementary video S1). We performed a double staining BrdU and Ki67 (Fig. 1a), a marker expressed in all dividing cells, and quantified the fraction of cells that left the cell cycle (immunolabeled BrdU+/Ki67− cells) relative to all BrdU incorporating cells [19] (BrdU+, Ki67− and BrdU+, Ki67+) in the occipital neocortex (i.e. the presumptive developing primary visual cortex) at E16.5 (Fig. 1a, b). At this stage, in mice the majority of retinal ganglion cells (RGCs) crosses the midline, projecting contralaterally [13]. Hence, the effect of drug-treatment is mirrored into the opposite brain hemisphere, whereas the ipsilateral cortices to the injected eye represent an internal control (untreated eye) (Fig. 1a, b). In addition, administration of BrdU in untreated animals at E15.5 and analyzed at P8 (when the various cortical layers occupy their final position) confirmed that the cortical neurons perturbed in our experimental setting belonged mainly to layer IV (Fig. 2; n = 3).

**Figure 3:**
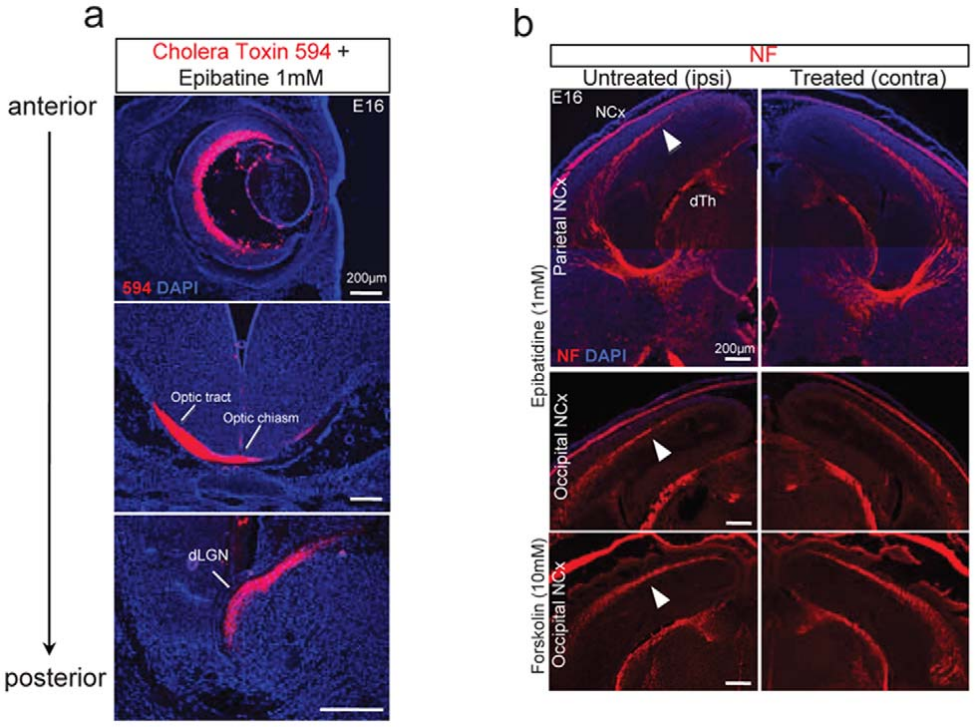
Timing and connectivity of retino-thalamic and thalamocortical visual pathways are established and not altered 12 h after ocular drug administration. (**a**) Representative images of antero-posterior coronal sections of the retina (top), optic chiasm (middle) and dLGN (bottom) after intraocular injection of Cholera Toxin conjugated with 594- red fluorophore and Epibatidine 1 mM (n = 3). The dye transport established connectivity between the retina and dLGN at E16. (**b**) Neurofilament (NF) staining showed an unaltered timing and connectivity of the thalamo-cortical pathway in E16 treated brains (n = 3 for each group). Abbreviations: dLGN dorsal lateral geniculate nucleus, dTh dorsal thalamus and NCx neocortex.

**Figure 4:**
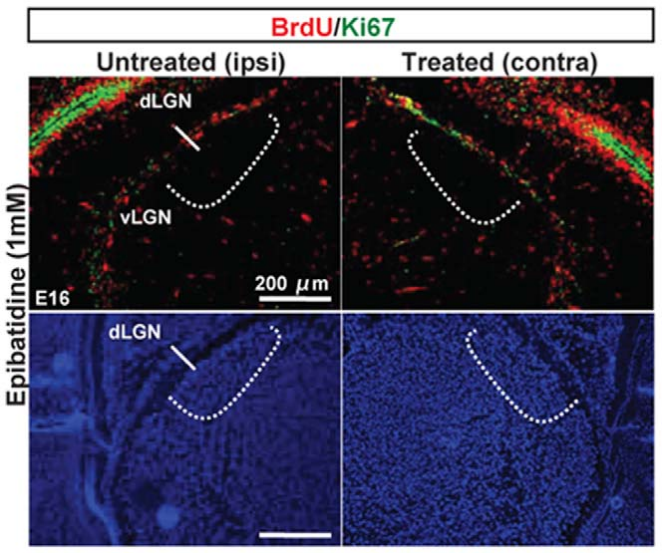
Ocular treatments do not impair neurogenesis in dLGN at E16. Representative images of Ki67 (green), BrdU (red) staining confirmed the absence of neurogenesis at E16 in the dLGN in treated animals. DAPI staining did not show structural anomalies in both contralateral and ipsilateral dLGN to the treated eyes.

Ocular epibatidine administration resulted in a significant 45% increase of cell cycle withdrawal (neurogenesis) in the cortices when compared to the internal opposite cortices (Fig. 1e; n = 6 animals). In addition, neurogenesis in the presumptive somatosensory area (S1) was equally distributed in both hemispheres (Fig. 1e) in treated groups, suggesting the specificity of the perturbation in V1. Injection of drug vehicle in the same litters did not show any differences (Fig. 1e; PBS n = 4 animals). These changes were not influenced by cell death, as assessed by TUNEL staining (data not shown). Stage I waves also have a Gap junction-mediated component refractory to pharmacologic cholinergic perturbation [9] and in addition, it has been observed that intraocular injection of epibatidine perturbs but does not block cholinergic-driven waves completely [20]. We thus performed acute monocular enucleation (ME) experiments to silence both components completely. E15.5 embryos were monocularly enucleated (ME) *in utero* (Fig. 1c, d, n = 4) and after 4 hours BrdU was administered. Similarly to epibatidine-treated animals 24 hours after ME (E 16.5) cortices contralateral to the enucleated eyes showed a 41% increase of neurogenesis restricted to V1 developing cortex compared to untreated animals (Fig. 1e). Cholera toxin tracing and neurofilament staining showed that connectivity of the retinogeniculate afferent and the geniculocortical radiation, respectively, are established at E16 and that treatments did not result in any apparent anomalies of these pathways as well as in the dLGN (Figs. 3 and 4). Although we did not directly measure the spontaneous firing characteristics resulting by epibatidine perturbation, these results allow inferring that the observed effect on cortical-neurogenesis by epibatidine treatment is owed to a complete and exclusive blockade of propagating cholinergic waves. However, considering that ME is a non-selective perturbation, we cannot rule out the possibility that distinct molecular cues may contribute to this phenomenon.

**Figure 5:**
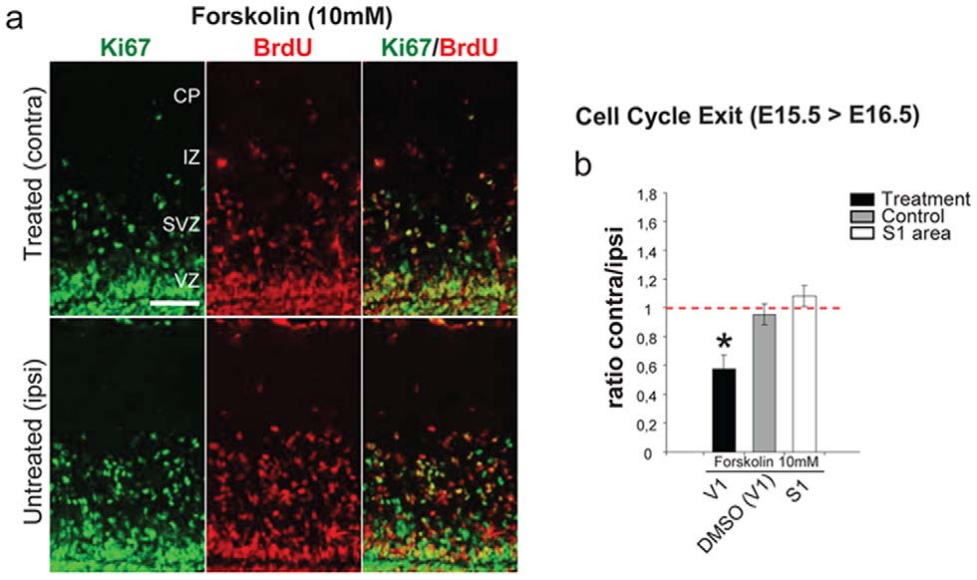
Foskolin treatment to the fetal retina decreases corticogenesis. Intravitreal (I.V.) injection of foskolin at E15.5 and BrdU and analysis in E16.5 embryos. (**a**) Representative images of Ki67 (green) and BrdU (red) labelling in contra-lateral and ipsi-lateral cortices after forskolin treatment. (**b**) Quantification of cell cycle exit rate, (ratio between contra- and ipsi-lateral cortices), in E16.5 embryos upon administration of forskolin decreases corticogenesis rate (forskolin n = 5 DMSO n = 6 p = 0.00077, Student's t-test, two samples equal variants). Abbreviations as for Fig. 1.

As a further support to the electrical nature of the signaling incoming from the retina to the developing dividing cortical neuroblasts, we injected murine fetuses monocularly at E15.5 with forskolin (cAMP activator; 10 mM), which has shown to increase the size, speed, and frequency of postnatal stage II cholinergic RWs firing pattern [21] driving an increased occurrence of spontaneous bursts in V1 [11], [12]. Although the effects of forskolin on Stage 1 waves are unknown, its presumptive increase of firing pattern in the developing retina, may complement the epibatidine experiment, further supporting the hypothesis that spontaneous activity drives the V1 effect observed. Strikingly, forskolin treatment was associated with a significant 40% decrease of newly born neurons (Fig. 5). Forskolin treatment alters cAMP levels, which is involved in many cellular processes. However, further studies are necessary to exclude that forskolin perturbs complex signaling besides activity [22]. Nonetheless, the biological complementary effect observed with epibatidine treatment supports a perturbation of the spontaneous firing pattern by forskolin treatment.

**Figure 6:**
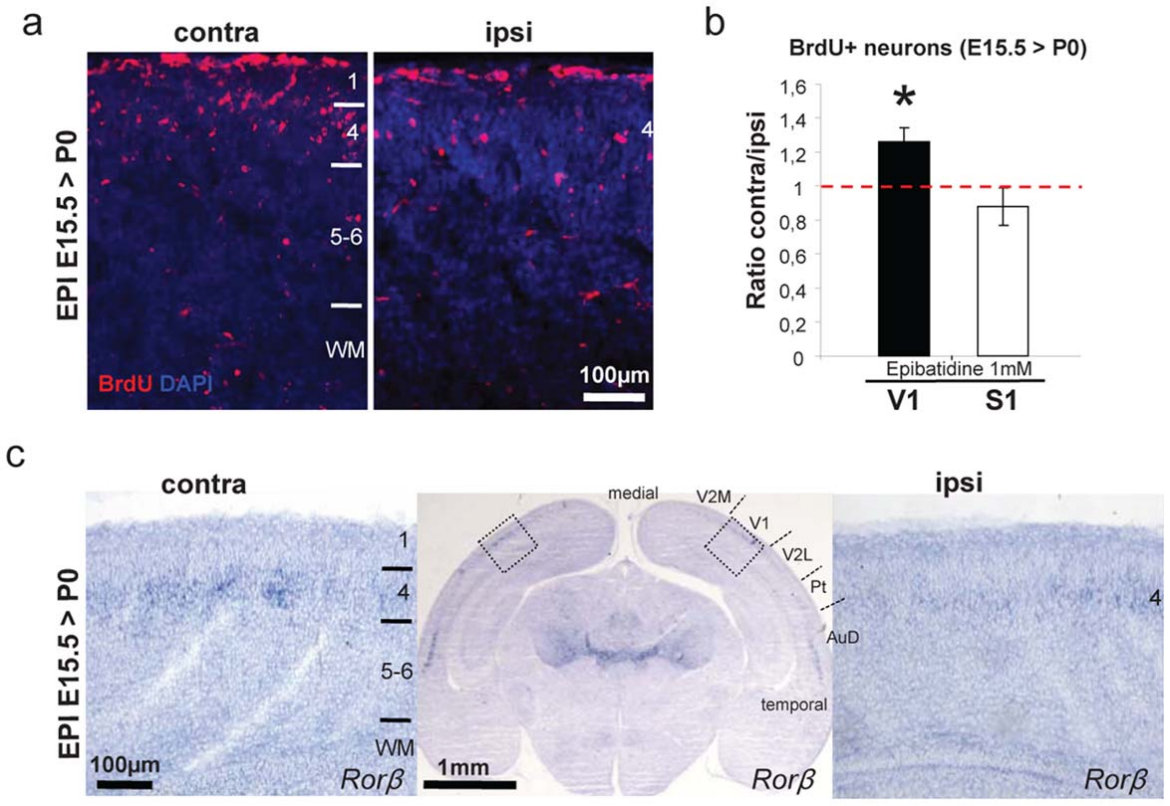
Embryonic retinal pharmacologic manipulation modifies cortical layer 4. (**a**) BrdU+ neurons in P0 cortices treated at E15.5 are increased in contralateral epibatidine-treated cortices. (**b**) Quantification of BrdU+ neurons after treatment with epibatidine (V1 vs S1 n = 4, p = 0.0072, Student's t-test, two samples equal variants). (**c**) Rorβ *in situ* hybridization in P0 animals treated at E15.5: layer 4 (IV) expression is increased in epibatidine treated compared to internal control cortices (ipsilateral; upper panels). Right and left panels represent a higher magnification of highlighted boxes in the middle panel (n = 6). Abbreviations: AuD auditory cortex, Pt parietal cortex, V1 primary visual cortex, V2L secondary visual cortex (lateral area), V2M secondary visual cortex (medial area) and WM, white matter.

To determine the effects of epibatidine at a later time point, we used the same protocol (pharmacological perturbation and BrdU administration) and sacrificed the animals at birth (E19, 4 days post treatment) (Fig. 6a, b). At this stage layer IV is localized at the top of the neocortical wall under the pial surface. Consistent with previous results, treatment with epibatidine resulted in 25% increase of BrdU+ cells (Fig. 6b, n = 4). Moreover, in P0 occipital cortices the specific layer IV marker Rorβ [23] was more specifically and intensively expressed in epibatidine treated animals (Fig. 6c; n = 6).

We found that the developing retina delivers signals to the cortex that are capable of regulating the very first developmental event of visual cortical development: cell genesis. This result suggests that the retina extrinsically coordinates the development of higher-order centers of visual representation well before visual-evoked patterns are generated. In addition, although we were unable to directly measure perturbations of retinal spontaneous activity, we found that the nature of this long-distance retinal to cortex signaling is consistent with retinal electrical spontaneous activity. This is supported by the following converging observations: i) inhibition of activity by cholinergic specific pharmacological blockade, ii) the high temporal resolution (24 hours) of perturbation of corticogenesis associated with the distance that needs to be covered (two orders of neurons coupled in series, such as ganglion and thalamo-cortical cells), and iii) phenotype complementation upon presumptive increase of activity provided with the cholinergic activator forskolin. In addition, the ME results further supports this finding. However, ME is a nonselective perturbation that may also abolish distinct molecular cues and may alter the LGN homeostasis (loss of growth factors, for instance). Nonetheless, both the converging results provided with pharmacological manipulations and the acute perturbation (24 hours, that unlikely can lead to major changes in the dLGN, as also observed by dLGN analysis) support the role of spontaneous activity in this phenomenon.

These results are consistent with studies showing a role of local electrical activity in regulating neurogenesis [24], [25]. Considering that the thalamo-cortical axon terminals invading the intermediate zone (IZ) and the SP of the developing cortex do not physically contact the proliferating neurons within the ventricular and subventricular zones (VZ - SVZ), these results imply that either directly an electrical field *per se* or a down-stream molecular secreted effector may eventually account for the effects on dividing neuroblasts. There is growing evidence that RWs act in combination with the expression of molecular cues such as ephrins [22], [26]. Notably, the ephrin/eph family is associated with corticogenesis [27]. An intriguing hypothesis is that the feature of spontaneous firing patterns contain key information that accordingly to their spatial (correlation) and temporal (structure) properties, activate a different set of genes leading to differential downstream effects. Future studies will be aimed at unraveling which are the temporal-spatial characteristics of RWs and their “instructive” or “permissive” interaction with molecular factors [28], [29].

These data show that retinal embryonic waves may represent a novel and robust extrinsic cue instructive to modulate cortical cell genesis in those neurons fated to become the main target of the retino-thalamic input. We conclude and propose that the embryonic retina through spontaneous activity delivers long-range information to its foremost distant terminal, the cortex, coupling the early coordinated development of the visual system as a whole.

## Supporting Information

Figure S1 **Methodology used for estimating cell cycle exit rate.** Representative “zoom in view” image of Ki67 (green), BrdU (red) labeling. White arrowheads depict cells withdrawn from the cell cycle as BrdU+ (red) cells, whereas yellow arrows cells reentering the cell cycle as BrdU+;Ki67+ (yellow) cells. The counted cells were used for estimating neurogenesis with the shown formula.
(TIF)

Video S1 **Drug intraocular injection in E15.5 embryos.** Exposed embryos after opening the uterus wall [16]. The needle tip is black painted enabling to track its exact position during drug intravitreal instillation. Drugs and vehicles were combined with 0.025% (w/v) fast green to evaluate the accuracy of the injection. Only mice successfully injected were considered for successive analysis.
(MP4)
