# Supplementary figures and images for: Mouse Embryonic Retina Delivers Information Controlling Cortical Neurogenesis
Source: PLoS One. 2010 Dec 8;5(12):e15211. doi: 10.1371/journal.pone.0015211 (PMC2999540; doi:10.1371/journal.pone.0015211)

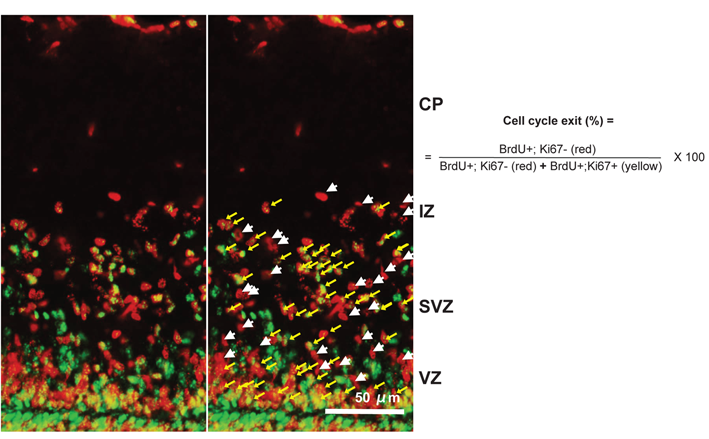

Supplement: Figure S1 — Methodology used for estimating cell cycle exit rate. Representative “zoom in view” image of Ki67 (green), BrdU (red) labeling. White arrowheads depict cells withdrawn from the cell cycle as BrdU+ (red) cells, whereas yellow arrows cells reentering the cell cycle as BrdU+;Ki67+ (yellow) cells. The counted cells were used for estimating neurogenesis with the shown formula. (TIF) [file pone.0015211.s001.tif]
